# 人源性的小细胞肺癌异种移植动物模型及耐药模型的建立

**DOI:** 10.3779/j.issn.1009-3419.2019.01.03

**Published:** 2019-01-20

**Authors:** 亚如 朱, 卫妹 黄, 源周 吴, 龙飞 贾, 雅玲 李, 瑞 陈, 琳琅 郭, 群清 陈

**Affiliations:** 1 510282 广州，南方医科大学珠江医院胸心外科 Department of Cardiothoracic Surgery, Zhujiang Hospital, Southern Medical University, Guangzhou 510282, China; 2 510282 广州，南方医科大学珠江医院病理科 Department of Pathology, Zhujiang Hospital, Southern Medical University, Guangzhou 510282, China

**Keywords:** 小细胞肺癌, 耐药, 人源性异种移植, 动物模型, B-NSGTM鼠, Small cell lung cancer, Drug resistance, Patient-derived xenotransplantation, Animal model, B-NSGTM mice

## Abstract

**背景与目的:**

小细胞肺癌（small cell lung cancer, SCLC）是细胞分化程度低，恶性程度高，生长速度快，早期容易发生转移的恶性肿瘤。目前SCLC患者的临床治疗以化疗为主，但是在治疗6个月-9个月后极易发生获得性耐药而复发。因此，构建有效的临床前SCLC动物模型具有重要的临床价值。人源性肿瘤异种移植动物模型（patient-derived xenotransplantation, PDX）能够较好地保留原发肿瘤的特性，是比较理想的临床前动物模型。本研究旨在构建中国人来源的SCLC PDX动物模型，并诱导构建化疗耐药的PDX模型，为研究SCLC耐药机制及个体化治疗提供实验模型。

**方法:**

取临床SCLC患者的新鲜手术切除标本或穿刺标本，移植至重度免疫缺陷小鼠NOD-PrkdcscidIL2rgtm1/Bcgen（B-NSGTM）皮下，HE染色及免疫组化对比移植肿瘤组织与患者肿瘤组织的病理学一致性。给予可稳定传代的每一代PDX模型小鼠腹腔注射8个周期的化疗药物（顺铂8 mg/kg+依托泊苷5mg/kg），定期监测荷瘤小鼠体质量和肿瘤体积，对长至1, 000 mm^3^大小的肿瘤进行传代移植。

**结果:**

本研究收集并移植9例SCLC肿瘤标本，成功构建可多次传代SCLC PDX模型3例并成功诱导其耐药模型，模型较好地保留了原发肿瘤的特征。

**结论:**

成功构建了SCLC PDX模型及其耐药模型，建模成功率为33%。为后续研究人的SCLC耐药机制、临床药物筛选以及个体化治疗提供了实验平台。

## Abstract (en)

**Background and objective:**

Small cell lung cancer (SCLC) is characterized by poor differentiation, high malignancy and rapid growth fast, short double time, early and extensive metastatic malignancy. In clinical, chemotherapy is the main treatment method, while resistance to multiple chemotherapy drugs in six to nine months has been a major clinical challenge in SCLC treatment. Therefore, It has important clinical value to building SCLC aninimal model which is similar to patients with SCLC. Animal model of xenotransplantation (PDX) from the patients with small cell lung cancer can well retain the characteristics of primary tumor and is an ideal preclinical animal model. The study is aimed to establish SCLC PDX animal model and induce the chemoresistance model to help to study the mechanism of chemoresistance and individual treatment.

**Methods:**

Fresh surgical excision or puncture specimens from SCLC patients were transplanted into B-NSGTM mice subcutaneous tissues with severe immunodeficiency in one hour after operation the B-NSGTM mice subcutaneous in 1 hour, and inject chemotherapy drugs intraperitoneally after its tumor growed to 400 mm^3^ with EP which is cisplatin 8 mg/kg eight days and etoposide 5 mg/kg every two days until 8 cycles. Measure the tumor volum and mice weights regularly, then re-engrafted the largest tumor and continue chemotherapy.

**Results:**

Nine cases were conducted for B-NSG mice modeling. Three of nine cases could be engrafted to new B-NSG mice at least two generation. The SCLC PDX animal models have been established successfully. After adopting chemotherapy drugs, the chemoresistance PDX models have been established. High homogeneity was found between xenograft tumor and patient's tumor in histopathology, immunohistochemical phenotype (Syn, CD56, Ki67).

**Conclusion:**

The SCLC PDX animal model and the chemoresistance PDX animal model have been successfully constructed, the success rate is 33%, which provides a platform for the clinical research, seeking for biological markers and choosing individual treatment methods of SCLC.

在全世界范围内肺癌是癌症患者死亡的首要原因，其中小细胞肺癌（small cell lung cancer, SCLC）占15%-18%^[1]^。SCLC是一种高代谢、高转移肿瘤^[2]^，其致死率高，5年生存率仅约5%。在临床上，EP方案（顺铂+依托泊苷）是SCLC最常用的化疗方案^[3]^。尽管大多数病人在初始治疗时对化疗药物敏感，但极易出现化疗耐药^[4, 5]^。因此，寻找SCLC有效的治疗手段，对延长SCLC患者的生存具有重要意义。人源肿瘤组织异种移植模型（patient-derived xenotransplantation, PDX）是指肿瘤患者的手术标本或穿刺标本不经过其他培养或处理而直接移植到免疫缺陷鼠皮下。相比于传统的细胞系来源的肿瘤动物模型（cell line derived xenografts, CDX），PDX模型很好的保留了肿瘤的异质性及遗传特性, 更符合临床病理特征^[6]^。PDX动物模型适用于评估临床药物的疗效，也可用于研究肿瘤的生物标记分子，还可以为个体化治疗研究提供实验手段^[7]^。目前国内外研究机构成功构建PDX模型的肿瘤有乳腺癌^[8]^、肝癌、胰腺癌^[9]^、食管癌^[10]^、胃癌^[11]^、结直肠癌^[12]^、宫颈癌、膀胱癌^[13]^、非小细胞肺癌^[14]^、胸膜间皮瘤^[15]^、头颈部鳞癌^[16]^、神经胶质细胞瘤^[17]^、小细胞肺癌^[18]^。由于SCLC早期容易发生转移，大部分病人在诊断时就已失去手术机会，临床标本难以获得，给PDX模型的构建带来了极大挑战。本研究成功构建3例人源化的SCLC荷瘤B-NSG鼠动物模型及其耐药模型，并可稳定多次传代于B-NSG鼠，移植瘤较好地保留了原发肿瘤的特性，为中国人SCLC的药物评估和筛选及患者的个体化治疗提供临床前研究平台。

## 1 材料与方法

### 1.1 材料

#### 1.1.1 肿瘤标本来源

SCLC肿瘤标本来自南方医科大学珠江医院、广东省人民医院和广州医科大学第二附属医院，标本的收集均取得患者知情同意，所有动物实验通过了南方医科大学和广州医科大学医学动物研究伦理委员会的批准。

#### 1.1.2 实验动物

动物实验所用小鼠NOD-PrkdcscidIL2rgtm1/Bcgen（B-NSGTM）均为雌性，4周龄-6周龄，体质量17 g-20 g，购于百奥赛图有限公司，实验动物合格证号：SCXK（苏）2016-0004。BALB/C鼠购于中山大学实验动物中心，全程饲养于无特定病原体（specific pathogen free, SPF）级动物实验房空气层流架内（室温恒定于20 ℃-22 ℃，空气湿度30%-50%），予以动物的饲料为钴辐射灭菌过的小鼠专用颗粒饲料，约每2天-3天更换清洁笼子。

#### 1.1.3 主要试剂

磷酸盐缓冲液PBS购自美国Gibco公司；利多卡因购自美国Sigma公司；顺铂注射液（诺欣）购自江苏豪森药业集团有限公司；依托泊苷注射液购自齐鲁制药（海南）有限公司；IHC试剂盒购于江苏凯基生物技术股份有限公司；突触素（synaptophysin Syn）、CD56即神经细胞粘附因子（neural cell adhesion molecule NCAM）、肿瘤增殖标志物Ki67的抗体试剂均购自于武汉赛维尔生物科技有限公司。

### 1.2 方法

#### 1.2.1 SCLC原代PDX动物模型的建立

收集SCLC患者手术标本3例及穿刺标本6例，放在盛有磷酸盐缓冲液（phosphate buffer saline, PBS）的清洁离心管中，用冰盒运送至动物中心后（组织保存时间30 min-60 min），立即用无菌器械把肿瘤分割成大小约0.3 cm×0.3 cm×0.3 cm的组织块。用70%酒精消毒小鼠右侧背部皮肤，在小鼠移植部位用0.5%利多卡因进行局部浸润麻醉，在小鼠右下肢背部用剪刀剪开长约0.3 cm的小口，把分割的肿瘤组织1块-2块送至皮下，按压约2 min，观察至小鼠切口粘合，以防肿瘤块脱出。此为原代PDX动物模型建立，称为P0代。

#### 1.2.2 SCLC PDX模型的传代移植

待P0代小鼠皮下瘤长至约1, 000 mm^3^时颈椎脱臼法处死小鼠，铺无菌巾，用70%酒精消毒小鼠背侧皮肤，用无菌器械切开瘤周围皮肤，剖出肿瘤放进无菌盘中，取部分组织置于4%中性甲醛溶液固定，剩余部分并用无菌器械分割肿瘤至大小约0.3 cm×0.3 cm×0.3 cm的组织块。取5只-10只5周龄B-NSG鼠，按照前述方法移植，此代为第1代PDX动物模型，称为P1代。每周定期检测小鼠的体质量及肿瘤的体积，绘制肿瘤生长曲线。待P1代B-NSG鼠皮下瘤长至大小约1, 000 mm^3^时，按照此方法传代移植，建立第2、3、4代PDX动物模型，称为P2、P3、P4代。小鼠皮下瘤体积计算方法为：肿瘤体积计算方法：V（cm^3^）=长（cm）×宽2（cm）/2^[18]^。

#### 1.2.3 诱导SCLC PDX耐药模型

待PDX动物模型P1代（每例8只B-NSG鼠）皮下肿瘤体积大小约400 mm^3^时，腹腔注射化疗药物（EP方案，顺铂8 mg/kg八天一次+依托泊苷5 mg/kg两天一次），连续注射8个周期后，挑选皮下肿瘤体积未明显减小的荷瘤鼠进行传代移植至P2（各例分别移植到5只BNS-G鼠），待其皮下瘤长至约400 mm^3^时，继续用EP方案腹腔注射药物8个周期。每一代移植设立对照组，对照组不用药物干预。

#### 1.2.4 生物学特性观察

小鼠一般特征观察每天观察精神状态、活动力、反应、饮食、体质量、毛色、毛顺滑度及皮下瘤的生长情况。在接种后1周，每7天用游标卡尺测量皮下瘤长、短径。大体解剖检查：颈椎脱臼处死小鼠并解剖，观察移植瘤形态、直径、质地、活动度。

#### 1.2.5 HE染色制备

将所取肿瘤组织用石蜡包埋，4 μm厚度连续切片，脱蜡脱水；入苏木素染液染5 min；水洗返蓝；依次入85%、95%的梯度酒精脱水各5 min后入伊红染液中染色5 min；无水乙醇脱水、二甲苯透明，封片，显微镜下观察并采集图片。

#### 1.2.6 免疫组化制备

依次将切片放入二甲苯两次各20 min、浓度为100%、95%、80%、75%梯度的酒精各10 min后置于蒸馏水；高温高压抗原修复；切片放入3%过氧化氢溶液，室温避光孵育20 min，将玻片置于PBS（pH7.4）中在脱色摇床上晃动洗涤3次，每次5 min；滴加一抗（Syn 1:200, CD56 1:200, Ki67 1:500）。切片平放于湿盒内4 ℃孵育过夜；玻片置于PBS（pH7.4）中洗涤3次，每次5 min。切片稍甩干后滴加二抗（HRP标记）覆盖组织，室温孵育50 min；玻片置于PBS（pH7.4）中在脱色摇床上晃动洗涤3次，每次5 min。切片稍甩干后滴加新鲜配制的DAB显色液，显色后自来水冲洗切片终止；苏木素复染3 min，水洗返蓝；将切片依次放入70%、80%、90%、95%酒精浓度梯度各5 min；无水乙醇脱水，二甲苯透明；中性树胶封片。

#### 1.2.7 免疫组化半定量分析方法

阳性细胞判断标准：Syn、CD56表达阳性为细胞浆着色，Ki67表达阳性为细胞核着色，阳性表达为浅黄色或棕黄色；免疫组化着色基于着色细胞百分比和强度：染色强度为0-3（0=阴性，1=弱阳性，2=中等强阳性，3=强阳性）。半定量计分系统H-score：（0×阴性细胞/100）+（1×弱阳性细胞/100）+（2×中等强阳性细胞/100）+（3×强阳性细胞/100），H-score得分范围为0-300。计算范围为光镜下小细胞肺癌的所有肿瘤细胞。

### 1.3 统计学处理

采用SPSS 19.0软件包处理，计数资料采用率（%）表示，组间比较采用χ^2^检验；计量资料采用均数±标准差（Mean±SD）表示，组间比较采用*t*检验，以*P* < 0.05为差异有统计学意义。

## 2 结果

### 2.1 SCLC PDX模型的建立及传代

本研究共收集并移植9例SCLC肿瘤标本，其中3例为手术标本，6例为穿刺标本。如图 1所示，第1号病例即#1，P0代成瘤率为20%（1/5）；P10%（0/5）。第2号病例即#2，P0代成瘤率为37.5%（3/8）；P1代成瘤率为83.3%（10/12）；P2代成瘤率为100%（10/10）。第3号病例即#3，P0代成瘤率为0%（0/5）。第4号病例即#4，P0代成瘤率为100%（2/2）；P1代成瘤率为0%（0/5）。第5号病例即#5，P0代成瘤率为80%（4/5）；P1代成瘤率为100%（10/10）；P2代成瘤率100（8/8）。第6号病例即#6，P0代成瘤率为100%（4/4）；P1代成瘤率为0%（0/10）。第7号病例即#7，P0代成瘤率为80%（4/5）；P1代成瘤率为80%（10/10）；P2代成瘤率100（7/7）。第8号病例即#8，P0代成瘤率为100%（2/2）。第9号病例即#9，P0代成瘤率为80%（4/5）。

**1:**
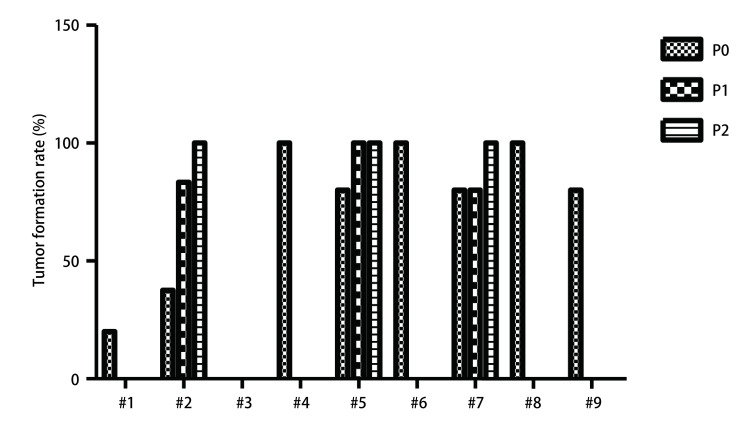
小细胞肺癌PDX动物模型成瘤率。PDX：patient-derived xenotransplantation P0：初次移植患者标本至B-NSG鼠皮下的代数。P1：P0代鼠皮下肿瘤移植至B-NSG鼠皮下的代数。P2:P1代鼠皮下肿瘤移植至B-NSG鼠皮下的代数 Tumor formation rate of SCLC PDX models. SCLC: small cell lung cancer.

### 2.2 PDX模型肿瘤生长曲线

移植后每7天测量皮下肿瘤长径及短径。各例患者标本所建动物模型P0代皮下瘤体积生长曲线见图 2。结果显示PDX动物模型皮下瘤在观察时间内P1代比P0代生长速度更快。如图 3，以第2例标本所建模型为例绘制P0、P1代肿瘤生长曲线（*P*=0.012，两者之间有统计学差异）。

**2:**
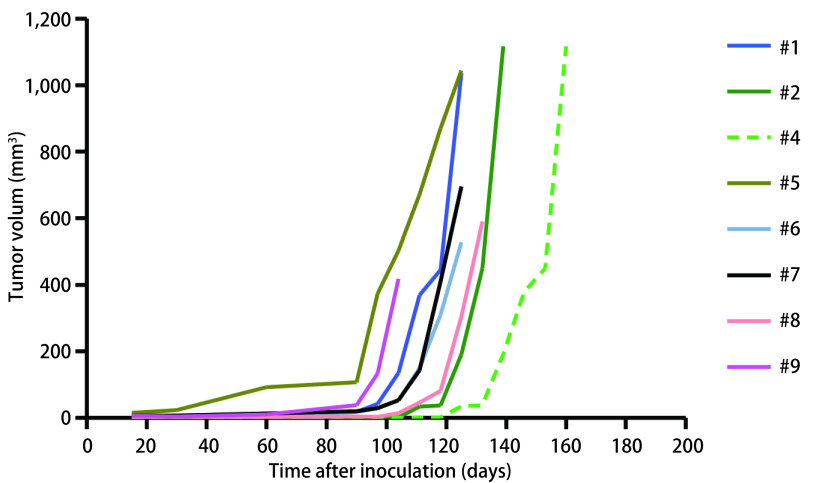
小细胞肺癌PDX动物模型P0代皮下瘤生长曲线 The growth curves of tumor in P0 of SCLC PDX models

**3:**
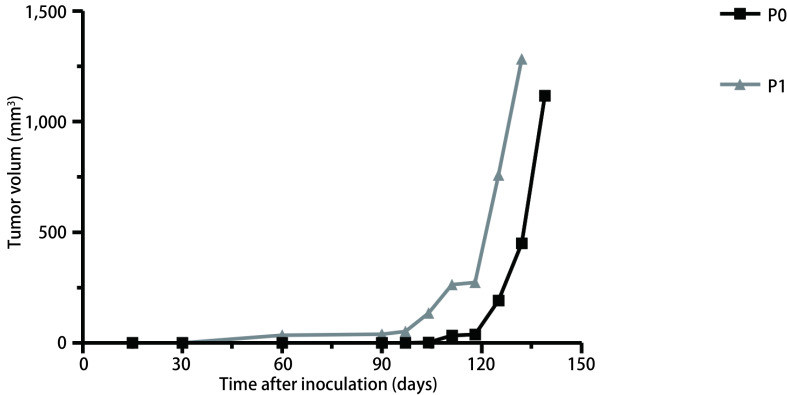
小细胞肺癌PDX动物模型P0、P1代鼠皮下瘤生长曲线（*P*=0.012） The growth curves of tumor in P0、P1 of SCLC PDX models (*P*=0.012)

### 2.3 诱导PDX化疗耐药模型

如图 4所示，分别以第2例患者（图 4A）、第5例患者（图 4B）肿瘤标本所建PDX动物模型诱导其耐药。分别给予P1代及P2代荷瘤B-NSG鼠注射化疗药物后测量肿瘤体积并绘制体积变化曲线。图 4A中示成功移植的P1代8只小鼠，其中3只小鼠肿瘤体积明显减小，2只小鼠死亡，另3只小鼠肿瘤体积减小后又增长。选择肿瘤体积最大的B-NSG鼠进行P2代移植，继续化疗药物处理后观察并绘制肿瘤体积变化曲线。图 4B示第5例患者来源肿瘤所建的PDX模型，P1代（*n*=4）及P2代（*n*=5）荷瘤B-NSG鼠给予化疗药物后存活率均为100%。结果表明随着化疗药物诱导的代数增加，PDX表现出更强的化疗抵抗性。图 4C简示PDX耐药模型的诱导流程。

**4:**
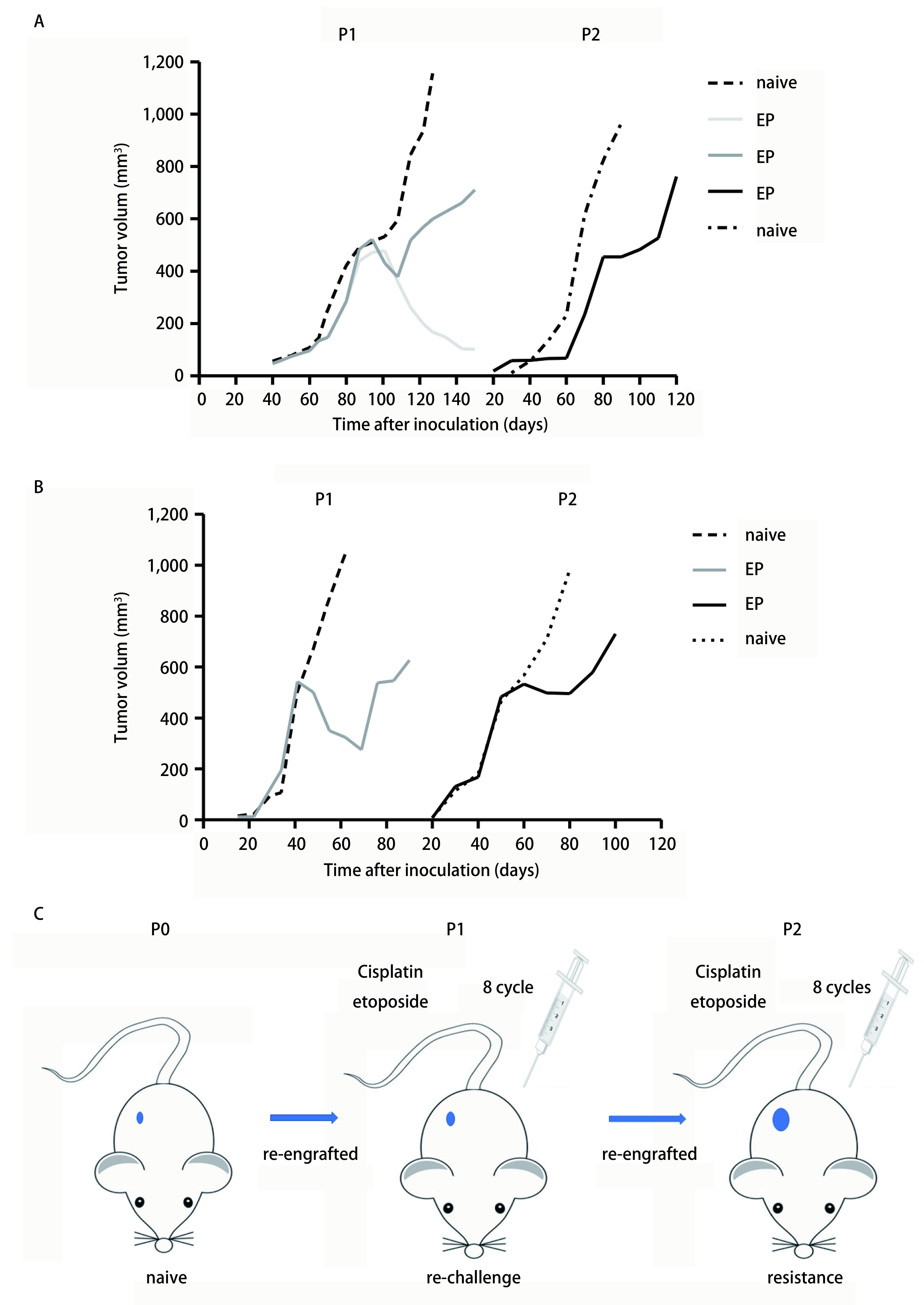
给予以2号、5号患者所构建PDX动物模型（图A、图B）荷瘤B-NSG鼠腹腔注射化疗药物后P1代、P2代B-NSG鼠皮下瘤的体积变化。A图P1代未化疗鼠*n*=5（虚线），化疗鼠*n*=8（实线）；P2代未化疗鼠*n*=3（虚线），化疗鼠*n*=5（实线）。B图P1代未化疗鼠*n*=5（虚线），化疗鼠*n*=5（实线）；P2代未化疗鼠*n*=3（虚线），化疗鼠*n*=5（实线）。C图构建小细肺癌PDX耐药模型模式图。 Changes of tumor volume in P1, P2 mice treated chemotherapy drugs (#2, #5). A: P1 naive *n*=5 (dotted), EP *n*=8 (solid line); P2: naive *n*=3 (dotted), EP *n*=5 (solid line); B: P1 naive *n*=5 (dotted), EP *n*=5 (solid line); P2: naive *n*=3 (dotted), EP *n*=5 (solid line). C: workflow of modeling acquired resistance C/E in SCLC. C/E: cisplatin and etoposide.

### 2.4 SCLC PDX模型肿瘤的实体照片

SCLC PDX动物模型建模成功后进行传代移植，图 5A示第2例患者来源的PDX模型P0肿瘤，图 5B示其P2代肿瘤。图 5C示P2代PDX模型化疗抵抗肿瘤（左）和化疗敏感肿瘤（右）。

**5:**
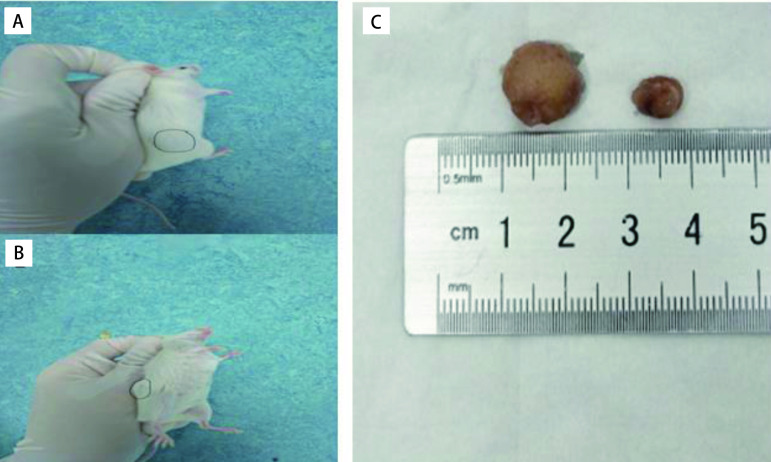
#2小细胞肺癌人源性异种移植长出肿瘤的小鼠及皮下瘤摘除实体照片。A：P0代荷瘤B-NSG鼠（*n*=3）；B：P2代荷瘤B-NSG鼠（*n*=10）；C：给予化疗药物后皮下瘤摘除照片（*n*=3）。 Images of SCLC PDX model and subcutaneous tumors of model #2. A: P0 tumor-bearing mouse (*n*=3); B: P2 tumor-bearing mouse (*n*=10); C: Picture of subcutaneous tumor after chemotherapy drug administration (*n*=3).

### 2.5 SCLC PDX动物模型皮下移植瘤大体形态

切开皮肤，保持皮下瘤完整，可见肿瘤表面光滑，可见清晰血管纹理，瘤整体呈红色或白色，瘤表面质韧，切开后，瘤中心呈乳白色，质软。

### 2.6 HE染色比较PDX肿瘤组织与患者肿瘤组织的病理形态

光镜可见可瘤细胞胞浆少，核大、深染，轻到中度异形，核仁不明显，呈巢状分布。PDX肿瘤组织与SCLC患者来源组织病理形态一致。以第2例患者为例见图 6。

**6:**
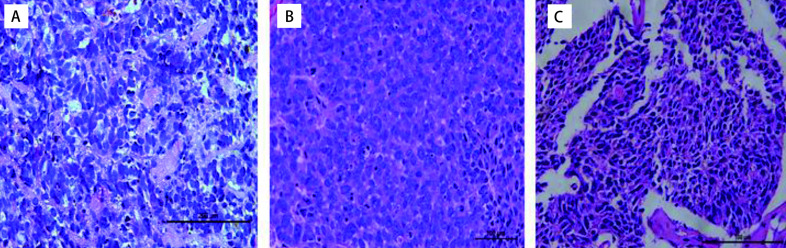
#2患者标本组织及小鼠皮下瘤组织结构（HE, ×400）。A：小细胞肺癌患者肿瘤组织；B：P0小细胞肺癌PDX动物肿瘤组织；C：P2代小细胞肺癌PDX动物肿瘤组织。可见瘤细胞胞浆少，核大、深染，轻到中度异形，核仁不明显，呈巢状分布。 The tissue structure of patient sample tissues and PDX model of model 2 (HE, ×400). A: Tumor tissue of small cell lung cancer patient; B: The tumor tissue in P0 of SCLC PDX model; C: The tumor tissue in P2 of SCLC PDX model. It can be seen that tumor cell cytoplasm is small, the nucleus is large, deeply infected, and light to moderate heteromorphism, the nucleoli are not obvious, and nest-shaped.

### 2.7 免疫组化比较PDX肿瘤组织与患者肿瘤组织的SCLC标记物表达

观察SCLC肿瘤标志物突触素（synaptophysin, Syn）、CD56及肿瘤增殖标志物Ki67在患者肿瘤组织及移植瘤组织的表达结果，可见肿瘤组织及各代PDX模型均阳性表达。如图 7所示以第2号病例患者肿瘤标本及移植瘤组织免疫组化图及H-score评分（每例标本做5张切片）。

**7:**
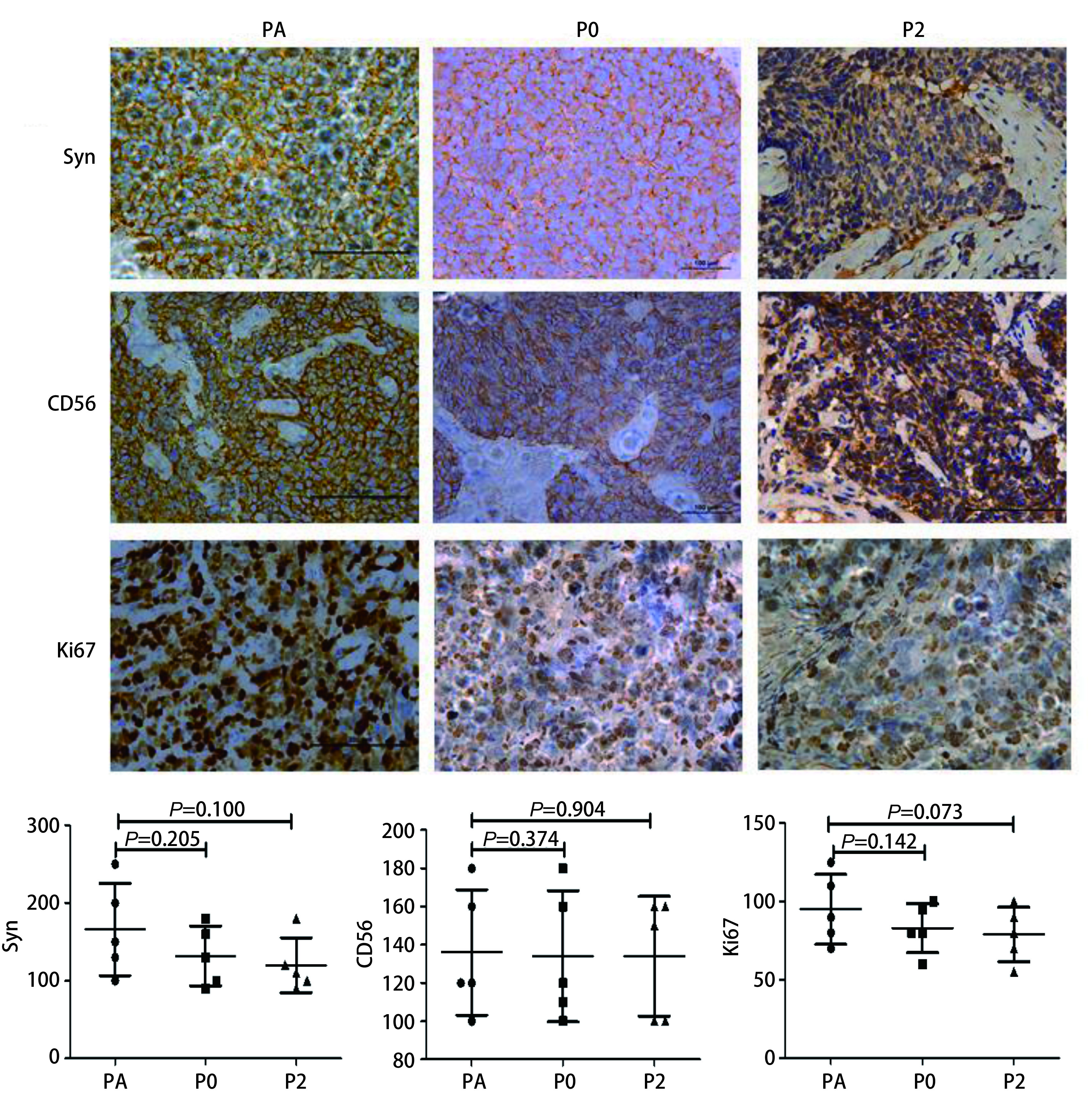
#2患者标本组织及PDX动物模型皮下瘤组织Syn、CD56、Ki67表达（×400）。#2患者肿瘤组织及PDX动物模型P0、P2代PDX肿瘤标志物CD56和Syn抗体以及肿瘤增殖标志物ki67表达阳性，每个样本5张组化片计分，H-score评分。*P* > 0.05，独立样本*T*检验，可见无明显差异。 Expression of Syn, CD56 and Ki67 in SCLC patient's tissues and the PDX tumor tissues of model #2 (×400). Expression of Syn, CD56 and Ki67 in SCLC patient's tissues and the PDX tumor tissues in P0, P2 mice of of model 2 were positive, H-score. *n*=5 per group. *P* > 0.05: Independent samples *t*-test; non-significant.

## 3 讨论

目前常用于研究肿瘤的动物模型有细胞源性异种移植（cell line-derived xenograft, CDX）模型和人源肿瘤异种移植（patient-derived xenograft, PDX）模型。CDX动物模型即把人来源的肿瘤细胞株注射到免疫缺陷小鼠皮下，用于评估化疗药物的疗效或进行肿瘤相关分子机制的研究。此种方法建立动物模型快捷，成瘤时间短，成瘤率高，在评估耐药机制方面有重大意义。但是CDX动物模型在临床结果预测方面具有局限性^[19]^。既往研究表明细胞培养传代时间越长，变异越大，和亲代肿瘤差异越大。Knudsen等^[20]^构建了54例胰腺癌PDX动物模型和56例CDX动物模型，发现CDX动物模型的肿瘤在建立和传代过程中会发生一定程度的基因突变。而在PDX动物模型的肿瘤基因高度保守，与亲代肿瘤相比基因遗传具有一致性，并可传至至少40代以上，基质细胞稳定性可保持3代以上。

PDX动物模型最早是Fiebig等^[21]^建成，Mattern等^[22]^应用于化疗药物的研究，发现PDX动物对生物碱类药物和抗代谢药物的反应和人相似。PDX动物模型避免了对优势克隆、表观遗传及基因修饰的自然选择，并保留亲代细胞的物种专一性及肿瘤细胞和基质细胞间的作用关系。PDX动物模型是直接从患者肿瘤标本接种到免疫缺陷鼠身上，未经过体外处理，避免了体外环境的选择，和CDX动物模型相比，与患者肿瘤特征更保持一致性，因此对药物疗效评估更接近临床效果^[23]^。Swick等^[24]^成功构建了27例头颈鳞癌的PDX模型，发现两者成瘤的组织学特性并无明显差别。Anderson等^[25]^用穿刺组织成功构建了SCLC PDX动物模型，并进行了HE及免疫组化检测，发现在组织学及化疗敏感性方面与亲代肿瘤相似。在本实验所建立的PDX动物模型，患者肿瘤组织和B-NSG鼠皮下移植瘤组织HE染色结果示两者无明显差异，可见瘤细胞胞浆少，核大、深染，轻到中度异形，核仁不明显，呈巢状分布，符合SCLC病理特点。SCLC又称小细胞神经内分泌癌，突触素（synaptophysin Syn）是位于突触前囊泡内的糖蛋白，存在于中枢和外周神经系统所有神经末梢，是目前诊断神经内分泌瘤的特异性标志物^[26]^。CD56即神经细胞粘附因子(neural cell adhesion molecule, NCAM)，它是一种跨膜糖蛋白, 属于免疫球蛋白超家族成员，在神经、神经外胚层、神经内分泌组织及其来源的肿瘤中可见特异性表达，在SCLC中的表达明显高于非小细胞肺癌^[27]^，因此CD56可作为SCLC肿瘤标志物之一。本研究中比较了Syn和CD56在SCLC患者肿瘤组织及PDX动物模型移植瘤组织中的免疫组化结果，结果可见均呈阳性，也观察了肿瘤增殖标志物Ki67在患者组织及移植瘤组织中的表达，可见呈阳性，说明PDX动物模型在免疫组化学方面保持稳定性，构建模型成功。

SCLC治疗早期对放疗及化疗很敏感，但是很容易出现耐药，因此，SCLC耐药是临床治疗的巨大瓶颈。本研究中按EP方案（顺铂8 mg/kg八天一次+依托泊苷5 mg/kg两天一次，腹腔注射）给予荷瘤B-NSG鼠化疗药物，注射8个周期后，对瘤体积最大的B-NSG鼠传代移植，继续按此方法注射化疗药物8个周期，可见皮下瘤体积增大，说明对化疗药物不敏感，出现耐药，构建SCLC耐药模型成功。把耐药组肿瘤及敏感组肿瘤分别移植到5只B-NSGTM小鼠，耐药组皮下成瘤时间为1个月，而敏感组为3个月，耐药组成瘤时间及皮下瘤生长速度均快于敏感组，表现出较高的侵袭性。成功构建SCLC耐药模型，为研究SCLC耐药机制及治疗方法提供良好平台。

Kubota等^[28]^在20世纪八九十年代最初报道了SCLC PDX动物模型，他们选择的是BALB/c免疫缺陷小鼠，发现尽管在宿主小鼠身上肿瘤细胞的动力学有些改变，但小细胞肺癌细胞的特性及总体参数仍具有稳定性。在本实验中发现移植小鼠的种类对小鼠成瘤率及生长情况有很大的影响，目前移植小鼠的种类有（1）裸鼠：不含T淋巴细胞，可能含有B淋巴细胞、组织粒细胞，含有自然杀伤细胞、树突状细胞的免疫缺陷小鼠。（2）SCID鼠（Severe combined immunodeficient mice）：不含T淋巴细胞及B淋巴细胞，含有自然杀伤细胞，组织粒细胞及树突状细胞。（3）NOD-SCID鼠：不含T淋巴细胞及B淋巴细胞，可能含有自然杀伤细胞，组织粒细胞及树突状细胞。与SCID鼠比较，NK细胞活性较低，免疫缺陷度较高。（4）B-NSGTM鼠（NOD.Cg-PrkdcscidIL2rgtm1Sug/Jic or NOD/Shi-scid IL-2Rγnull）：NOD-SCID遗传背景，敲除IL-2rg，缺乏成熟的T细胞、B细胞、NK细胞，免疫缺陷度更高。在本实验中选择B-NSGTM重度免疫缺陷小鼠构建SCLC PDX动物模型，相较BALB/c鼠，B-NSGTM重度免疫缺陷小鼠成瘤时间为3个月-4个月，肿瘤生长速度快。另在移植过程中发现，移植部位对成瘤也有影响。我们发现移植在靠近右下肢部位更容易成瘤，考虑此部位比背部血液供应丰富，肿瘤组织更容易生长。但此部位会受小鼠活动影响，致使刚移植的肿瘤组织脱出，成瘤后也影响小鼠活动。目前我们选择的移植部位是右侧背部，受小鼠活动影响较小，即小鼠活动不会造成肿瘤组织脱出，肿瘤长大后不会影响小鼠活动。

SCLC为高度侵袭性肿瘤，大部分患者确诊已处于晚期，失去手术治疗时机，构建模型主要通过穿刺收集少许组织标本。标本量少增加了PDX动物模型构建的难度，也极大限制了对SCLC发病和耐药机制的研究，本研究中动物模型成瘤率为33%，构建SCLC PDX动物模型及耐药模型具有重要意义，为SCLC的药物研究评估及筛选、SCLC患者的个体化治疗以及SCLC耐药机制、研究靶向药物提供良好的模型。
